# Ultrasound Stimulation Increases Neurite Regeneration in Injured Dorsal Root Ganglion Neurons through Mammalian Target of Rapamycin Activation

**DOI:** 10.3390/brainsci10070409

**Published:** 2020-06-30

**Authors:** Sungmin Han, Jinyoung Park, Won Seok Choi, Inchan Youn

**Affiliations:** 1 Biomedical Research Institute, Korea Institute of Science and Technology, 5, Hwarang-ro 14-gil, Seongbuk-gu, Seoul 02792, Korea; cws0104@kist.re.kr; 2 Molecular Recognition Research Center, Korea Institute of Science and Technology, Hwarangno 14-gil 5, Seongbuk-gu, Seoul 02792, Korea; jypark@kist.re.kr; 3 Division of Bio-Medical Science &Technology, KIST School, Korea University of Science and Technology, 5, Hwarang-ro 14-gil, Seongbuk-gu, Seoul 02792, Korea

**Keywords:** ultrasound stimulation, neurite regeneration, mammalian target of rapamycin, dorsal root ganglion

## Abstract

Ultrasound stimulation (US) is reported to be a safe and useful technology for improving injured nerve regeneration. However, the intracellular mechanisms underlying its stimulatory effects are only partially understood. Mammalian target of rapamycin (mTOR) signaling is involved in neuronal survival and axonal outgrowth. In this study, we investigated the effect of US on regeneration of injured dorsal root ganglion (DRG) neurons and activation of the mTOR pathway. We showed that US significantly increased neurite regeneration and enhanced mTOR activation. Moreover, the expression of growth-associated protein-43 (GAP-43), a crucial factor for axonal outgrowth and regeneration in neurons, was significantly increased by US. These data suggest that US-induced neurite regeneration is mediated by upregulation of mTOR activity, which promotes the regeneration of injured DRG neurons.

## 1. Introduction

Nerve injury is commonly caused by accidental trauma and results in disconnection and disruption of axonal pathways, leading to permanent physiological impairments, such as paralysis and loss of sensation [1,2]. Whereas the central nervous system (CNS) has a limited ability for repair and regeneration, resulting in permanent damage after injury, axons in the peripheral nervous system (PNS) can spontaneously regenerate following injury [3,4]. The regenerative capacity of the PNS is supported by a variety of extrinsic and intrinsic factors; axons and myelin sheaths of denervated nerves are degraded by Wallerian degeneration, and neurons switch to a regenerative state to promote axonal regeneration and reorganization [5]. However, the functional recovery of peripheral nerves following clinical injury is highly limited and unsatisfactory. For example, a lack of directional organization of injured axons might cause failure of targeted organ reinnervation. Delayed axonal regeneration can result in functional impairments despite nerve continuity. Therefore, increasing the speed and efficacy of axonal regeneration is important for achieving successful regeneration after peripheral nerve injury.

Various physical stimulation methods were assessed to determine whether they can improve peripheral nerve regeneration, such as lasers [6,7], magnetic fields [8], and electricity [9]. Promising results regarding the ability of these methods to accelerate axonal regeneration were achieved, but these methods present challenges; they are unable to penetrate deep targeted tissues or localize changes due to their insufficient spatial resolution, and they require additional surgery for electrical component implantation. Recently, ultrasound (US) stimulation received considerable attention for its ability to enhance peripheral nerve regeneration. US is a noninvasive stimulation method and can deliver acoustic energy that induces various biological effects in the targeted tissue. For example, low-intensity pulsed US with a fundamental frequency (FF) of 1.5 MHz leads to earlier recovery of myelin sheaths after neurotomy and thus nerve regeneration [10]. An FF of 1 MHz accelerates peripheral nerve regeneration by directly stimulating conduits seeded with Schwann cells to bridge sciatic nerve defects in rats [11,12]. Jiang et al. explored the effects of different US intensities on reversal transplantation of autologous nerve grafts and identified the optimal intensity of US for improving regeneration of damaged nerves in an experimental rat model [13]. These studies suggest that US can promote injured peripheral nerve regeneration, but little is known about the mechanism via which US affects the response of neurons. 

Therefore, this study investigated whether US can promote peripheral neurite regeneration and regulate regeneration-mediated signaling pathways. Neurites of dorsal root ganglion (DRG) neurons were severed directly above a cultured DRG explant to establish a peripheral injury model. US was applied to stimulate the DRG neurons, and neurite regeneration was assessed by comparing the numbers and lengths of regenerating neurites. Mammalian target of rapamycin (mTOR) plays a crucial role in the regulation of cellular growth and survival [14]. To explore the effect of US on the mTOR signaling pathway, the expression of upstream regulators of mTOR, including phosphatase and tensin homolog (PTEN) and protein kinase B (Akt), and mTOR activation in response to US were evaluated. We showed that US significantly promoted neurite regeneration and mTOR activity. Moreover, growth-associated protein-43 (GAP-43) expression was increased by US. These results suggest that US-induced mTOR activation can increase the expression of GAP-43, thereby enhancing neurite regeneration of injured peripheral neurons.

## 2. Materials and Methods

### 2.1. US Stimulation Set-Up

**Figure 1:**
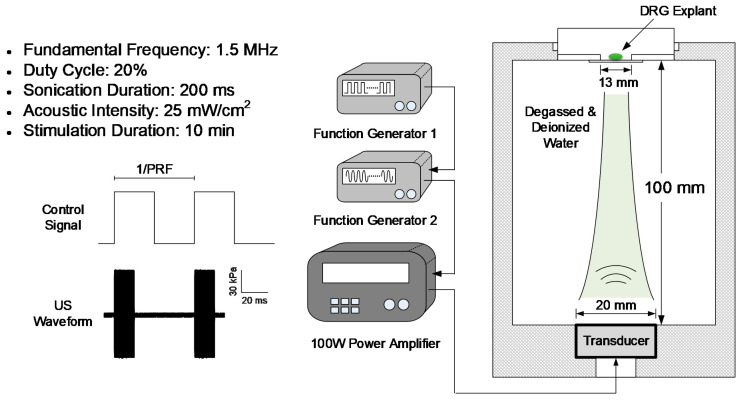
Experimental set-up for ultrasound stimulation (US). The dorsal root ganglion (DRG) explant was positioned 100 mm above the transducer. The chamber was filled with degassed and deionized water. The acoustic stimulation parameters were set to a fundamental frequency of 1.5 MHz, a sonication duration of 200 ms, a stimulation duration of 10 min, and an acoustic intensity of 25 mW/cm^2^.

Figure 1 shows the experimental set-up used for US stimulation of DRG explants. US was generated using an unfocused piezo-ceramic flat transducer (DONG IL Technology, Ltd., Gyungyido, Korea) with an aperture diameter of 20 mm and an FF of 1.5 MHz. Pulsed sine signals were induced by two serially connected waveform generators and then amplified by a 100-Watt linear radio-frequency power amplifier (AG 1021, T&C Power Conversion, Inc., Rochester, NY, USA). The transducer was positioned at the bottom of a custom-made acrylic water chamber filled with deionized and degassed water, and the culture dish was located 100 mm perpendicularly above the transducer based on the natural focal depth, which was calculated using the following formula:

(1) $$\mathrm{Natural}\;\mathrm{focal}\;\mathrm{depth}\;\left(\mathrm{mm}\right)=\frac{D^{2}\times f_{0}}{4c}$$

where *c* is the speed of sound in the medium, *D* is the aperture diameter, and $f_{0}$ is the FF. A 13-mm-diameter hole was located in the center of the bottom of the culture dish, and the hole was covered with 130-μm-thick glass; this 130-μm glass was much thinner than the ultrasound wavelength at 1.5 MHz FF.

Acoustic pressure was measured in a water tank filled with degassed and deionized water using a calibrated needle hydrophone with an aperture of 500 μm (HNR-500, Onda Corporation, USA) as described in a previous study with some modifications [15]. The hydrophone tip was located 100 mm from and parallel to the geometric center of the transducer based on the experimental set-up. The acoustic intensity (AI) was represented as the spatial-peak temporal-average intensity (Ispta), which was calculated according to a method described by a previous study [16]. The density and speed of the medium were assumed to be 1028 kg/m^3^ and 1515 m/s, respectively. The acoustic output parameters were set to an FF of 1.5 MHz, a sonication duration (SD) of 200 ms, and a duty cycle (DC) of 20%. US was applied to the DRG explants for 10-min periods immediately after axotomy.

### 2.2. DRG Explant Culture

**Figure 2:**
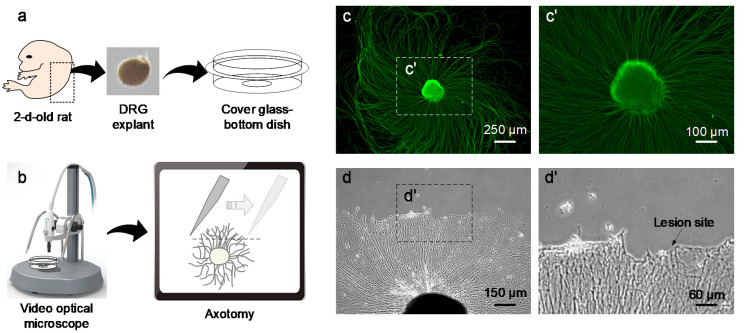
Representative images of cultured dorsal root ganglion (DRG) explants. (**a**) Schematic of the DRG explant culture. (**b**) Schematic of the axotomy method. (**c**) β-Tubulin fluorescence image of a DRG explant after five days of incubation. (**d**) Transacted neurites of DRG explants by axotomy. (c’, d’) Higher magnification images of the inserted boxes in (**c**,**d**).

All experiments were performed in accordance with the care and use of laboratory animals protocols approved by the Institutional Animal Care and Use Committee of Korea Institute of Science and Technology (2020-053). Figure 2a shows a schematic of the method used for DRG explant culture, which was performed according to a previous report with some modifications [9]. DRGs were obtained from two-day-old male Sprague-Dawley rats (Samtako, Osan, Korea). The rats were sacrificed by decapitation without anesthesia, and then the vertebral columns were separated. Under a dissection microscope (Olympus SZ51; Olympus Optical, Tokyo, Japan), the DRGs were carefully divided from the spinal cord using scissors and fine forceps, and nerve fragments and residual tissues were removed. The isolated DRGs were transferred to a 35-mm-cover glass-bottom dish (confocal dish, SPL Life Sciences, Pocheon, Korea) precoated with 10 μg/mL poly-d-lysine (Millipore, Bedford, MA, USA) and 100 μg/mL laminin (Gibco Invitrogen, Grand Island, NY, USA). The culture medium consisted of 2% B-27 supplement (Gibco Invitrogen), 1% penicillin–streptomycin–glutamine (Gibco Invitrogen), and neurobasal medium (Gibco Invitrogen). To inhibit the proliferation of mitotic cells, 10 μM/mL cytosine arabinoside (Sigma-Aldrich, St.Louis, MO, USA) was added to the culture medium for five days of incubation. The medium was changed every two days, and the cultured DRG explants were incubated at 37 °C with 5% CO_2_ in air.

### 2.3. Injured DRG Explants by Axotomy

After five days of incubation, axotomy was performed by transecting the neurites of DRG explants. DRG explants with neurites three times longer than the DRG explant diameter were used [9]. Damage to and detachment of the neuronal cell bodies and proximal neurites of the lesion were avoided to allow successful neurite regeneration. Figure 2b shows a schematic of the axotomy method, which was performed based on our previous study [9]. To prevent detachment of the neuronal cell body and the proximal neurites of the injured site, one side of the neurites was carefully separated from the culture dish using a fine pin under 100× magnification through a video optical microscope system (SV-35, SomeTech, Seoul, Korea). The lesion was approximately 400–500 μm from the DRG explant body, and the fragments of distal neurites were stripped from the culture dish.

### 2.4. Assessment of Neurite Regeneration

A total of 12 DRG explants were used, and the numbers and lengths of regenerating neurites were evaluated in each of four DRG explants. One day after axotomy, neurite regeneration of each axotomized DRG explant was analyzed by capturing graphic image files using a phase-contrast microscope (Olympus CX 40; Olympus Optical, Tokyo, Japan) and a microscope camera controller (Digital Sight DS-U3; Nikon Instruments, Japan). In each of the four DRG explants (*n* = 4), the numbers and lengths of regenerating neurites were analyzed with NIH image software (ImageJ, Bethesda, MD, USA) to evaluate neurite regeneration. The length of the longest neurites was determined as the distance between the processes extending from the edge of the lesion and the 10 longest neurite tips as previously described [9]. In the same images, the number of regenerating neurites was measured in a rectangular box of 300 × 300 μm located at the injury site above the center of the DRG explant body. The investigators were blinded to the treatment assignments.

### 2.5. Immunohistochemistry

Immunohistochemistry was performed based on a previous study [17]. Briefly, DRG explants were fixed in 4% paraformaldehyde in phosphate-buffered saline (PBS, Lonza, Walkersville, MD, USA) for 30 min at room temperature and then washed three times for 2 min each with PBS. Then, 0.2% Triton X-100 in PBS was applied for 20 min to achieve cell membrane permeabilization at room temperature, and the explants were washed again. Nonspecific binding was blocked by incubation with 4% bovine serum albumin (BSA, Millipore) in PBS for 1 h at 4 °C. DRG explants were washed three times with PBS and incubated with primary mouse monoclonal β-III tubulin antibody (Abcam, Cambridge, UK) overnight at 4 °C. A secondary antibody conjugated with Alexa Fluor 488 goat anti-mouse immunoglobulin G (IgG, Invitrogen) was applied to the DRG explant for 1 h at room temperature. After several washes with PBS, the DRG explants were observed using a fluorescence inverted microscope (Nikon Eclipse TS 100; Nikon Instruments) equipped with a charge-coupled device (CCD) digital camera (CoolSNAP^TM^ HQ2, Photometrics, Tucson, AZ, USA) and a mercury lamp (Intensilight C-HGFI, Nikon Instruments, Tokyo, Japan). Green fluorescent protein was viewed with a Semrock Brightline^TM^ single-band filter set optimized for fluorescein isothiocyanate (FITC) (FITC-A-Basic-NQF; Semrock Inc., Rochester, NY, USA). Images were recorded using the open-source Micro Manager 1.4 software add-on in ImageJ. The experimenters were blinded to the treatment assignments.

### 2.6. Western Blot Analysis

To examine the production of proteins, harvested cells were lysed in lysis buffer (iNtRon Biotechnology, Korea) and homogenized. The prepared lysates were boiled in 5× SDS sample buffer, and equal amounts of all cell lysates were loaded onto 8% or 12% sodium dodecyl sulfate polyacrylamide gels and transferred to polyvinylidene fluoride membranes (Bio-Rad, Hercules, CA, USA). The membranes were blocked with 5% BSA solution in Tris-buffered saline (20 mM Tris/HCl, pH 7.6, 150 mM NaCl, and 0.1% Tween 20) for 1 h and incubated overnight with primary antibodies at 4 °C. p-PTEN (1:1000, Cell Signaling Technology, Danvers, MA, USA), PTEN (1:1000, Cell Signaling Technology), glyceraldehyde 3-phosphate dehydrogenase (GAPDH) (1:1000, Abcam), p-protein kinase B (Akt) (1:1000, Cell Signaling Technology), Akt (1:1000, Cell Signaling Technology), p-mTOR (1:1000, Cell Signaling Technology), mTOR (1:1000, Cell Signaling Technology), and GAP-43 (1:1000, Abcam) primary antibodies were used. The membranes were incubated for 1 h with a horseradish peroxidase (HRP)-conjugated goat anti-rabbit IgG secondary antibody (Novus Biologicals, Littleton, CO, USA) at room temperature and then treated with enhanced chemiluminescence solution (Thermo Fisher Scientific, Waltham, MA, USA). Images of the bands were obtained using a chemiluminescence imaging system (iBright CL750, Thermo Fisher Scientific), and the relative intensity value of each protein band was calculated as the mean intensity of three DRG explants (*n* = 3).

### 2.7. Statistical Analysis

Statistical analyses were performed using SPSS software (SPSS 15.0, SPSS Science, Chicago, IL, USA), and data are presented as the mean and standard error of the mean (mean ± SEM). Differences between groups were compared using one-way analysis of variance with Tukey’s multiple comparisons test, and values with *p* < 0.05 were considered significantly different. 

## 3. Results

### 3.1. US Increases Regeneration of Injured Neurites

To analyze the effect of US on neurite regeneration, DRG explant neurites were grown to a length that allowed axotomy, and morphological changes and neurite regeneration were observed. Figure 2c shows a representative fluorescence microscopic image of a DRG explant. The DRG explant with neurites could be clearly identified and grew in all directions after five days of incubation. DRG explants with neurites that were three times longer than the diameter of the DRG explants after five days of culture were used in this experiment.

**Figure 3:**
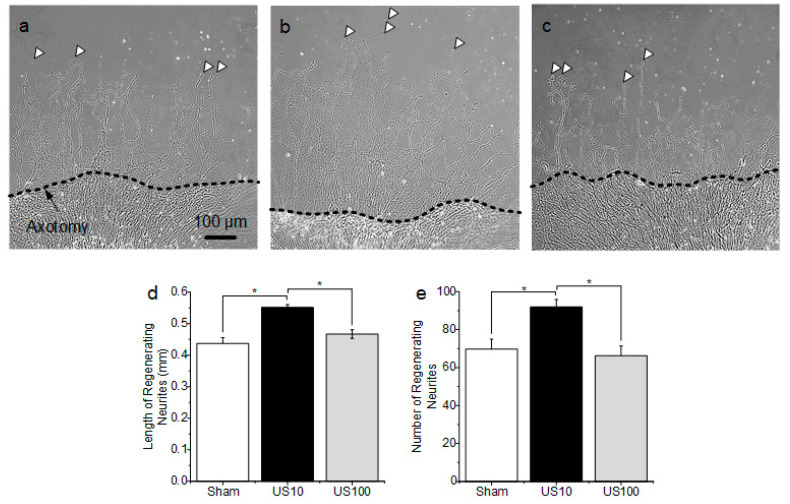
Evaluation of neurite regeneration of axotomized dorsal root ganglion (DRG) explants in response to ultrasound stimulation (US). (**a**) Representative images of regenerating neurites not treated with US (sham group). (**b**) Representative images of regenerating neurites treated with 10-Hz pulse-repetition frequency US (US10 group). (**c**) Representative images of regenerating neurites treated with 100-Hz pulse-repetition frequency US (US100 group). (**d**) Comparison of the lengths of regenerating neurites (*n* = 4). (**e**) Comparison of the numbers of regenerating neurites (*n* = 4). The data are expressed as the mean ± standard error of the mean (SEM); * *p* < 0.05.

Figure 2d shows an example of an injured DRG explant by axotomy in which the distal neurites were completely removed from the injury site and the proximal neurites were well attached. To evaluate the effect of US on regeneration of injured DRG explants with neurites, the numbers and lengths of regenerating neurites induced by two different US parameters were compared after one day of US stimulation. Representative images of neurite regeneration in the non-US (sham) group, a group treated with a pulse-repetition frequency (PRF) of 10 Hz (US10), and a group treated with a PRF of 100 Hz (US100) are presented in Figure 3a–c, respectively. The US10 group exhibited significantly longer regenerating neurites than the sham or US100 group. Regenerating neurites in the US10 group were approximately 26.18% (*p* = 2.334 × 10^−7^) and 18.18% (*p* = 1.112 × 10^−6^) longer than those in the sham and US100 groups, respectively (Figure 3d), but the difference between the sham and US100 groups was not significant (*p* = 0.195). Moreover, the number of regenerating neurites in the US10 group was significantly increased by 31.90% (*p* = 0.014) and 38.87% (*p* = 0.007) compared with those in the sham and US100 groups, respectively (Figure 3e), but the difference between the sham and US100 groups was not significant (*p* = 0.649). These results demonstrate that US can improve neurite regeneration in axotomized DRG explants, and that US10 stimulation effectively promoted neurite regeneration in our experiment. Therefore, US10 stimulation was used for all subsequent experiments.

### 3.2. PTEN and Akt Are Not Affected by US

**Figure 4:**
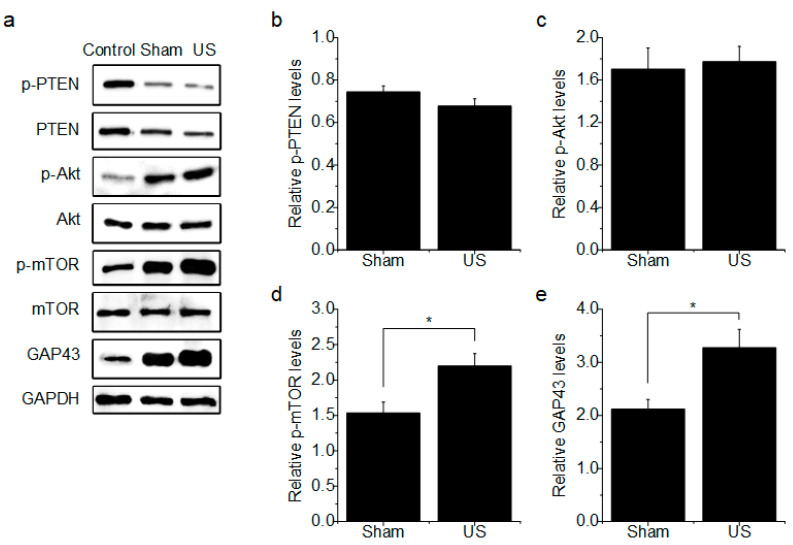
Protein expression of phosphatase and tensin homolog (PTEN), protein kinase B (Akt), mammalian target of rapamycin (mTOR), and growth-associated protein-43 (GAP-P43) in axotomized DRG explants. (**a**) Representative Western blot image. (**b**) Relative p-PTEN levels in the sham and US groups (*n* = 3). (**c**) Relative p-Akt levels in the sham and US groups (*n* = 3). (**d**) Relative p-mTOR levels in the sham and US groups (*n* = 3). (**e**) Relative GAP-43 levels in the sham and US groups (*n* = 3). The data are presented as the mean ± SEM; * *p* < 0.05.

Injured PNS neurons are required to activate a specific and orchestrated sequence of signaling pathways related to the regeneration program, and PTEN and Akt are key members of the phosphatidylinositol 3-kinase (PI3K) pathway, important for the regulation of neuronal survival and growth. We examined the effect of US on the expression and activation of PTEN and Akt in axotomized DRG explants. Figure 4a shows representative immunoblot results. The intensity of the specific bands was standardized to that of the GAPDH band. The expression levels of p-PTEN/PTEN and p-Akt/Akt were compared (Figure 4b,c). p-PTEN expression was decreased by axotomy (~0.74-fold), and p-Akt expression was increased (1.65-fold). PTEN was shown to be an inhibitor of the PI3K pathway and acts as an intrinsic obstacle to axonal regeneration in peripheral nerve lesions [18]. Akt is a downstream target of PI3K signaling and is upregulated by decreased PTEN activation after injury [14]. Therefore, injured DRG neurons might be expected to downregulate PTEN activation and elevate Akt phosphorylation to enhance regenerative capacity. The expression of p-PTEN was decreased by US (~0.68-fold), and p-Akt expression was increased (~1.68-fold); however, the difference between the sham and US groups was not significant (p-PTEN/PTEN: Sham vs. US, *p* = 0.201; p-Akt/Akt: Sham vs. US, *p* = 0.793). These results indicate that US does not affect the expression or activation of PTEN or Akt in axotomized DRG neurons.

### 3.3. US Increases mTOR Activation and GAP-43 Expression

The mTOR pathway plays an important role in numerous cellular processes through regulation of protein synthesis [14]. To determine whether US increases mTOR activity in axotomized DRG explants, the expression and the activation of mTOR were investigated. The expression level of p-mTOR/mTOR was compared, and mTOR phosphorylation was increased by 1.5-fold and 2.2-fold in the sham and US groups, respectively. However, p-mTOR expression was significantly increased in the US group compared to the sham group (Figure 4d) (*p* = 0.037). Next, the expression of GAP-43 was compared between the sham and US groups. GAP-43 was increased by 2.1-fold and 3.2-fold in the sham and US groups, respectively, and GAP-43 expression was significantly increased in the US group compared to the sham group (Figure 4e) (*p* = 0.031). These results suggest that US increases mTOR activation and GAP-43 expression in axotomized DRG explants. 

## 4. Discussion

Peripheral nerve repair remains a challenge in severe injury, and successful regeneration is one of the greatest clinical interests. Injured neurons require complex cellular and molecular mechanisms to regenerate successfully, and understanding the regeneration process is key to proposing new therapies for nerve repair. This study evaluated effective US parameters to enhance neurite regeneration in injured DRG neurons. As shown by the results, US10 treatment significantly improved the numbers and lengths of regenerating neurites in axotomized DRG explants. Moreover, US significantly increased mTOR activity and GAP-43 expression. These observations indicate that US leads to enhanced neurite regeneration in injured DRG explant neurons as a result of increased mTOR activity.

US is a mechanical acoustic wave at a frequency above the range of human hearing (approximately 20 kHz), and it is emerging as a novel therapeutic tool for clinical treatment due to its noninvasiveness, high spatial resolution, deep penetration, and cost-effectiveness. Therapeutic US intensities differ depending on the intended effect. High-intensity US can destroy target tissues through thermal or mechanical changes induced by high levels of acoustic energy delivered to a specific area [19]. Low-intensity US can potentially induce biological effects such as neuromodulation, tissue regeneration, bone-fracture healing, and anti-inflammatory responses [20]. Previous studies investigated US modalities to induce therapeutic changes in peripheral nerve injury. For example, the application of US to implanted conduits seeded with Schwann cells improves regeneration by increasing the number and area of myelinated axons [11]. US treatment accelerates the regeneration and functional recovery of neurotometically injured sciatic nerves in rats [10,21]. Autograft peripheral nerve regeneration in rats was improved by US, and an AI of 250 mW/cm^2^ significantly accelerated axonal regeneration [13]. These studies revealed that US can promote peripheral nerve regeneration, but the biological mechanisms underlying the effects of US stimulation are only partially understood. The current study investigated whether US can improve neurite regeneration in DRG explants injured by axotomy, which were used as a model of peripheral nerve injury to observe changes in signaling pathways during neurite regeneration [9]. US significantly increased both the number and the length of regenerating neurites, and mTOR activity was elevated by US. These results demonstrate that US-induced upregulation of the mTOR pathway may enhance neurite regeneration in axotomized DRG explants.

Various stimulation parameters, such as the FF, PRF, SD, AI, and tone-burst duration (TBD), may play key roles in achieving the desired effects of US [16]. With regard to the choice of PRF, superior neurite regeneration was observed when a PRF of 10 Hz was used compared to a PRF of 100 Hz. We do not know the exact reason for this observation, but we speculate that 10 Hz might have caused the recruitment of more excitatory neural circuits than 100 Hz. Our previous paper showed that US can directly stimulate neuronal activity through calcium channel activation in cortical neurons [15]. Calcium signaling might be involved in the activation of molecular mechanisms and post-translational processes on gene expression leading to the production and formation of a new growth cone and axon [22,23,24]. In addition, mTORC1, one of the mTOR complexes, is activated by extracellular calcium influx [25,26], which may be an important factor in mTOR activation by US. Further study is needed to reveal the detailed mechanism of US in the modulation of neurons.

To achieve successful regeneration of damaged nerves, neurons firstly detect injury signals and then initiate intrinsic mechanisms to control regenerative programs and promote axonal regeneration. The PI3K pathway is well known to promote cell survival and growth, and it is regulated by key proteins, including PTEN, Akt, and mTOR [14]. PTEN acts as a negative regulator, where it antagonizes PI3K activity by converting phosphatidylinositol (3,4,5)-trisphosphate (PIP_3_) to phosphatidylinositol (4,5)-bisphosphate (PIP_2_), thereby downregulating Akt. Akt phosphorylation is increased by PI3K activation and subsequently promotes target protein translation by modulating mTOR activity [27]. Many studies indicated that the PI3K pathway is involved in enhancing axonal outgrowth in developing and regenerating neurons. PTEN constrains the intrinsic regenerative capacity of the nervous system, and inhibition of PTEN expression enhances optic nerve regeneration and increases mTOR activity. In addition, blocking mTOR activity with rapamycin, a specific pharmacological inhibitor of mTOR, dramatically prevents axonal growth in retinal ganglion neurons [28]. PTEN inhibition also accelerates neurite outgrowth in sensory neurons and axonal outgrowth following sciatic nerve transection injury [18]. Although PTEN inhibition facilitates the axonal outgrowth of both central and peripheral neurons, this signaling pathway differentially regulates downstream effectors of mTOR. PTEN inhibition facilitates the growth of central neurons altered by mTOR activation [18]. mTOR activity contributes to boosting the regenerative potential of PNS neurons after damage, but neurite outgrowth following PTEN inhibition in peripheral neurons is independent of mTOR [29,30]. GAP-43 is a downstream target of mTOR activity, and it is associated with axonal sprouting and outgrowth in regenerating axons [29,31]. Tuberous sclerosis complex 2 (TSC2) knock-out neurons exhibiting increased mTOR activity showed enhanced GAP-43 levels in both naïve and injury-induced conditions. In addition, treatment with rapamycin partially blocked the increase in GAP-43 levels after injury, showing that the mTOR pathway regulates GAP-43 expression. Since GAP-43 plays an important role in axon sprouting and outgrowth in regenerating axons, regulation of GAP-43 expression by mTOR can be considered to contribute to improvement of the axon growth ability through the mTOR pathway. Thus, the mTOR pathway might regulate the translation of a number of proteins, including GAP-43, to maximize the axonal growth capacity [29]. In this study, US significantly increased both mTOR activity and GAP-43 expression in axotomized DRG explants. From these data, we believe that US of injured DRG explant neurons can contribute to enhancing in mTOR activity, thereby increasing GAP-43 expression and neurite regeneration.

Although our results suggest that US has a positive effect on neurite regeneration in axotomized DRG explants by regulating mTOR activity, other upstream or downstream regulators of mTOR specifically activated by US must be identified in future studies. As shown in Figure 4, US did not affect the expression or activation of PTEN or Akt, which are main regulators of mTOR activity in axotomized DRG neurons. Therefore, we want to identify other regulators involved in changes in mTOR signaling induced by US. The candidate may be TCS1/2, which regulates the upstream activity of mTOR, or a downstream factor such as eukaryotic initiation factor 4E-binding protein (4E-BP) and phosphorylation of ribosomal protein S6 kinase 1 (S6K1), which are affected by changes in mTOR activity induced by US. mTOR can be activated through phosphorylation and inactivation of TCS1/2. TCS1/2-mediated mTOR activation leads to phosphorylation of its substrate 4E-BP or S6K1, resulting in upregulated translation of a large pool of proteins in the regenerative process, such as GAP-43 [32]. Based on this possibility, we can confirm the phosphorylation of TCS1/2 and substrates by performing a Western blot assay when US is applied to injured neurons. Additionally, we will be able to observe the effect of reducing the expression of TCS1/2, 4E-BP, or S6K1 using small interfering RNA (siRNA) targeting each candidate on the activity of mTOR and the expression of GAP-43. More details about the signaling pathway associated with US-induced regeneration will be investigated in the future.

## Abbreviations

US	Ultrasound stimulation
DRG	Doral root ganglion
mTOR	Mammalian target of rapamycin
TEN	Phosphatase and tensin homolog
Akt	Protein kinase B
GAP-43	Growth-associated protein-43
FF	Fundamental frequency
Ispta	Spatial-peak temporal-average intensity
PRF	Pulse-repetition frequency
AI	Acoustic intensity
SD	Sonication duration
DC	Duty cycle
TSC	Tuberous sclerosis complex
4E-BP	Eukaryotic initiation factor 4E-binding protein
S6K1	Phosphorylation of ribosomal protein S6 kinase 1
